# Advance directives and end-of-life care: knowledge and preferences of patients with brain Tumours from Anhui, China

**DOI:** 10.1186/s12885-020-07775-4

**Published:** 2021-01-05

**Authors:** Yixin Wang, Yongkang Zhang, Yang Hong, Ping Zeng, Zongtao Hu, Xiuli Xu, Hongzhi Wang

**Affiliations:** 1 Department of Oncology, Hefei Cancer Hospital; Chinese Academy of Sciences, Hefei, 230031 Anhui China; 2 Anhui Provincial Key Laboratory of Medical Physics and Technology,Center of Medical Physics and Technology, HeFei Insititutes of Physical Science, Chinese Academy of Sciences, Hefei, 230031 Anhui China; 3 University of Science and Technology of China, Hefei, 230026 China; 4 Department of Neurosurgry, The First Affliated Hospital of Anhui Medical University, Hefei, 230022 China

**Keywords:** Advance directives, End-of-life care, Patients with brain tumours

## Abstract

**Background:**

In Mainland China, advance directives (ADs) and end-of-life care for patients with tumours, especially patients with brain tumours who may have lost consciousness or the ability to speak at the early stage of their illness, have been poorly acknowledged. Thus, this study aimed to clarify the knowledge and preferences of ADs and end-of-life care in patients with brain tumours and to investigate predictors of patient preferences.

**Methods:**

This was a population-based cross-sectional survey that was conducted via face-to-face interviews. Information on sociodemographic factors, brain tumour illness, knowledge and preferences of the advanced decisions and end-of-life care of the patients was collected.

**Results:**

A total of 88.61% of participants had never heard of ADs, but 65.18% reported that they would like to make ADs. Knowledge of ADs, receiving surgical treatment or radiotherapy, being younger than 70 years old, being male, having educational qualifications of college or beyond, being childless, having medical insurance for nonworking or working urban residents and self-paying medical expenses were predictors of preference for making ADs. A total of 79.43% of participants wanted to discuss end-of-life arrangements with medical staff, and 63.29% of participants were willing to receive end-of-life care, even though it would not delay death. A total of 65.82% of patients with brain tumours wanted resuscitation, and as many as 45.45% of the patients thought that they did not need life support if they were in a persistent vegetative state. Brain primary tumours, being younger than 70 years old, male sex, educational qualification of junior middle school or below, having children, having new rural cooperative medical insurance and having medical expenses paid by children or spouses were predictors of choosing appropriate palliative care.

**Conclusions:**

ADs and end-of-life care have been poorly acknowledged among patients with brain tumours in mainland China. Additional efforts should be encouraged amongst patients with primary brain tumours, those who are undergoing surgery and radiotherapy and those who have low socioeconomic status. A longitudinal and comprehensive study is encouraged to promote disease-specific ADs among Chinese patients with brain tumours.

**Supplementary Information:**

The online version contains supplementary material available at 10.1186/s12885-020-07775-4.

## Background

‘Advance directives (ADs)’ is the legal term for the documentation of end-of-life care decisions that honour an individual’s values and preferences [1, 2]**.** In mainland China, advance directive discussions are challenging and complicated, and no relevant legal document exists; such a situation has an inevitable and important effect on patients with tumours because patients with cancer who convey their individual values and end-of-life decisions are more likely to receive care that they want than those who do not convey their values and decisions [3]. ADs help patients fully understand end-of-life decision-making to generate an appropriate therapeutic schedule that may reduce chemotherapy and targeted medicine use and decrease hospitalizations [4]. Moreover, ADs allow patients with tumours to spend meaningful time with their families and die with dignity. Therefore, oncology medical staff should investigate ADs for tumour patients. In particular, in contrast to patients with other types of cancer (e.g., lung cancer) who may be consistently conscious, patients with brain tumours may lose consciousness or their ability to speak during the early stage of their illness. Thus, implementing ADs for patients with brain tumours is important. However, few studies have concentrated on the knowledge and preferences of ADs in Chinese patients with tumours, especially patients with brain tumours. In this work, the primary aim was to describe the knowledge and preferences of ADs and end-of-life care decisions of patients with brain tumours. The secondary aim was to investigate the roles played by disease-related factors, sociodemographic characteristics and economic conditions in predicting the preferences of participants.

## Methods

### Participants

Patients from Cancer Hospital, Chinese Academy of Science, Hefei and The First Affiliated Hospital of Anhui Medical University were eligible if they were greater than 18 years old, with confirmed brain tumour diagnosis, no disturbance of consciousness, the capacity to understand and talk to clinical staff and were assessed by clinical staff to be physically and mentally able to complete the questionnaire.

### Study design

Follow-up was difficult given that patients with brain tumours have poor prognosis and may reside in rural areas. Thus, a cross-sectional study was conducted from January 1 to February 20, 2020. All patients with brain tumours who met the inclusion criteria were interviewed, among whom 44 were unwilling to be interviewed. Ethical approval of the research protocol (SL-KY2020–002) was granted by the Ethics Committee of the Cancer Hospital of the Chinese Academy of Science, Hefei.

### Data collection procedure

Briefly, potentially eligible patients were identified by clinical staff from the clinician workstation. Considering that some patients’ family members usually asked the doctor to keep the patients’ conditions confidential, we introduced our study to the patient’s family members before we interviewed the patients. We provided the following information: *Our study is a face-to-face interview. Patients will be asked some questions about ADs (a written statement of a person’s wishes regarding medical treatment that often includes a living will and is made to ensure that these wishes are carried out should the person be unable to communicate them to a doctor), which is not legal at present, and end-of-life care (support and medical care given during the time surrounding death).* Then, we asked family members two questions: *(1) ‘Does the patient know the true condition of their illness?’(2) ‘Will you allow the patient to participate in this interview?’*

If we had the permission of family members and the patients understood the true condition of their illness, we conducted our interview. The questionnaire (Additional file 1) was formulated in accordance with the literature on ADs and end-of-life care. All data were collected by our research team through face-to-face interviews in the patients’ ward. Considering that not all patients with brain tumours could read, we read the questions aloud and recorded the patients’ answers.

### Questions about advance directives

Patients were asked to respond to the following statement: (1) ‘*Have you heard of AD (a written statement of a person’s wishes regarding medical treatment, often including a living will, made to ensure that these wishes are carried out should the patient be unable to communicate them to a doctor)?’ (2) ‘In the future, if AD become legal in China and you had a condition wherein you could not make your own medical decisions (e.g., comatose or could not speak), would you like to make an AD?’ (3) ‘If you prefer to make an AD, your reasons will be to a) ensure that the end of my life is comfortable and avoid pain; b) avoid imposing economic burden on my family; c) hope my own wishes will be respected; d) avoid imposing burdens on society; e) consider that the quality of my life is more important than the length of my life; f) religious beliefs and g) I have witnessed the rescue of other people.’ (4) ‘Your reasons for not preferring to make an AD are a) my family members will decide for me; b) I will let it be; c) I have no need to think about it now and my doctor will decide for me; d) my decision may change; e) I am not familiar with the concept of advance directives and f) religious beliefs.’*

### Questions about end-of-life care

First, the concept of end-of-life care was introduced for patients as follows: *end-of-life care cannot cure your illness. It is care given to people who are near the end of their life and have stopped treatment to cure or control their disease. End-of-life care includes physical, emotional, social and spiritual support for patients and their families. The goal of end-of-life care is to control pain and other symptoms such that the patient can be as comfortable as possible. End-of-life care may include palliative care, supportive care and hospice care*. They were then asked to respond to the following statements: *(1) ‘Would you like the medical staff to discuss your illness and end-of-life arrangements directly with you?’ (2) ‘If you were terminally ill (a few weeks before death), would you prefer to receive appropriate palliative care that cannot delay death but provides comfort?’ (3) ‘If you were terminally ill (a few weeks before death) and in critical condition (e.g., cardiac arrest), would you prefer receiving resuscitation (cardiopulmonary resuscitation, electrical defibrillation, endotracheal intubation or tracheotomy)?’ (4) ‘If you were terminally ill (a few weeks before death) and in a persistent vegetative state (such as brain tumour progression), would you prefer life support (including nutritional support, such as tube feeding or percutaneous endoscopic gastrojejunostomy, broad-spectrum antibiotics, blood transfusions or ventilator assisted ventilation?’*

### Sociodemographic and disease variables

Information about the patients, including sociodemographic factors, type of brain tumour, history of brain tumours and general condition, was recorded.

### Data analysis

Data were analysed using SPSS 22.0 (IBM Corp, Armonk, NY). The relationships among the participants’ sociodemographic factors, history of brain tumours, preference for making ADs and preference for receiving appropriate palliative care were analysed via binary logistic regression. All *p*-values less than 0.05 were considered statistically significant. Group comparisons were determined by two questions about ADs and end-of-life care.

## Results

### Characteristics of study participants

Sociodemographic characteristics of the 316 participants with brain tumours who were greater than 18 years old are reported in Table 1. Among the patients, 65.19% were female, and 60.12% were male. In terms of marital status, approximately two-thirds of the participants were married, 15.83% were divorced, 11.39% were unmarried and 8.86% had lost their spouses. Over 70% of participants completed junior high school education or below, and only 24 had a bachelor/postgraduate/PhD degree. Although only 17.21% of participants were urban residents, 76.9% had their own private housing. Moreover, the type of medical insurance of the participants was related to their residence: 82.28% of patients had new rural cooperative medical insurance, 22.71% had medical insurance for nonworking urban residents and only 6.01% had medical insurance for working urban residents.

**Table 1:** Characteristics of Brain Tumor Patients(*n*=316)

Social and Demographic Characteristics	n(%)
**Age,years**	
30–39	18 (5.69%)
40–49	20 (6.32%)
50–59	88 (27.84%)
60–69	97 (30.69%)
70–79	72 (22.78%)
80–89	21 (6.64%)
**Sex**	
Male	110 (34.81%)
Female	206 (65.19%)
**Marital status**	
Married	202 (63.92%)
Divorced	50 (15.83%)
Unmarried	36 (11.39%)
Widowed	28 (8.86%)
**Educational Qualifications**	
Junor middle school or less	229 (72.47%)
Senior high school	63 (19.94%)
College or more	24 (7.59%)
**Residence**	
Rural	260 (82.29%)
City	56 (17.21%)
**Type of housing**	
Private housing	243 (76.9%)
Retal housing	22 (6.96%)
Children’s housing	51 (16.14%)
**Religious belief**	
None	234 (74.05%)
Have	82 (25.95%)
**Children**	
None	61 (19.3%)
Have	255 (80.7%)
**Type of medical insuranse**	
New rural cooperative medical insurance	260 (82.28%)
Medical insurance for non-working urban residents	37 (11.71%)
Medical insurance for working urban residents	19 (6.01%)
**Who will cover your medical expenses?**	
Own	110 (34.81%)
Children or spouse	206 (65.19%)

Tumour-related characteristics of the 316 participants are reported in Table 2. Over two-thirds of participants had brain metastases, among which primary tumours were located in the lung (32.27%) and breast (35.75%). Nearly a quarter of primary brain tumours were classified as gliomas. Only three patients had meningioma, and one patient had brain lymphoma. At the time of the interview, 73.74% of participants were receiving radiotherapy, 8.22% were receiving surgery and 14.55% were receiving medicine. Only 11 participants were undergoing support treatment. Slightly more than one-half of participants had been ill for less than 12 months, and 18.68% of participants had lived for over 25 months. Only 26.27% of patients with brain tumours were in good general condition with KPS scores over 80. Over 70% of patients with brain tumours had poor KPS scores less than 80 and/or even less than 50.

**Table 2:** General Condition of Brain Tumor Patients (*n*=316)

Morbid Characteristics	n(%)
**Type of brain tumor**	
Glioma	78 (24.68%)
WHO I-II	14 (4.43%)
WHO III-IV	64 (20.25%)
Meningioma	3 (9.49%)
Lymphoma	5 (1.58%)
Brain Metastases	
From lung cancer	102 (32.27%)
From breast cancer	113 (35.75%)
From other cancer	15 (4.73%)
**Treatment currently receiving**	
Surgery	26 (8.22%)
Radiotherapy	233 (73.74%)
Medicine	46 (14.55%)
Support treatment	11 (3.49%)
**Duration of illness**	
1 month or less	81 (25.63%)
2 months to 6 months	32 (10.13%)
7 months to 12 months	54 (17.08%)
12 months to 24 months	90 (28.48%)
25 months or more	59 (18.68%)
**Symptoms**	
Headache	181 (57.27%)
epilepsy	21 (6.65%)
Movement disorders	78 (24.69%)
Language disorders	36 (11.39%)
**KPS scores**	
<50	37 (11.71%)
60–79	196 (62.02%)
>80	83 (26.27%)

*KPS scores* Karnofsky scores

### Advance directives

The knowledge and preferences of patients for ADs are summarized in Table 3. A very low proportion of patients with brain tumours had ever heard of ADs, while 88.61% had never heard of ADs. After introducing the concept of ADs to patients, almost two-thirds of participants stated that they would like to make ADs. As shown in Table 3, the primary reason for wanting ADs was to ensure comfort at the end of their lives and reduce financial burdens on their family. For those who would not like to make ADs, the key reason was their lack of familiarity with the concept of ADs or their belief that doctors (30.91%) or family members would make decisions for them.

**Table 3:** Knowledge and preferences of the study participants regarding ADs (*n* = 316)

Questions	Answers	n%
Have you heard of ADs (a written statement of a person’s wishes regarding medical treatment, often including a living will, made to ensure these wishes are carried out should you be unable to communicate them to a doctor)?	Yes	36 (11.39%)
	No	280 (88.61%)
In the future, if ADs were legal in China and you were in a condition wherein you cannot make your own medical decisions (e.g. in coma or cannot speak), would you like to make an AD?	I would	206 (65.18%)
	I would not	110 (34.82%)
**Reasons for preferring to make an AD**		
Ensure that the end of my life is comfortable and avoid pain		93 (45.15%)
Avoid burdening my family’s economic condition		66 (32.03%)
Hope my wishes could be respected		23 (11.17%)
Avoid imposing burdens on society		3 (1.45%)
Consider the quality of my life is more important than the length of my life		19 (9.23%)
Religious beliefs		0
Witnessed the rescue of other people		2 (0.97%)
**Reasons for preferring not to make an AD**		
Family members will decide for me		15 (13.64%)
Let it be		5 (4.55%)
No need to think about it now and my doctor will decide for me		34 (30.91%)
Decision may change		6 (5.45%)
Not familiar with the concept of ADs		47 (42.73%)
Religious beliefs		3 (2.72%)

*ADs* Advance directives

### Predictors of preferring to make advance directives

Bivariate analyses identified knowledge of ADs, receiving surgery or radiotherapy, age of less than 70 years old, male sex, educational qualification of college or beyond, without children, medical insurance for nonworking or working urban residents and self-payment of medical expenses as significant predictors of preferring to make ADs. Table 4 shows the results of the binary logistic regression model.

**Table 4:** Binary logistic regression model prediction of preference for making ADs (*n* = 316)

Independent Predictors	OR	95%CI	*p* value
**Heard of ADs**			
Yes	4.727	1.626–13.745	0.04*
No (reference group)	1		
**Current treatment**			
Surgery	34.5	4.918–242.028	< 0.001*
Radiotherapy	14.223	2.985–67.778	0.001*
Medicine	1.25	0.232–6.739	0.795
Support treatment (reference group)	1		
**KPS scores**			
< 50	2.426	0.951–6.190	0.064
60–79	0.954	0.560–1.626	0.862
> 80 (reference group)	1		
**Age in years**			
30–49	4.756	1.971–11.476	0.001*
50–69	3.424	2.031–5.773	< 0.001*
70–89 (reference group)	1		
**Sex**			
Male	3.224	1.861–5.586	< 0.001*
Female (reference group)	1		
**Educational qualifications**			
Junior middle school or less (reference group)	1		
Senior high school	0.848	0.428–1.503	0.572
College or beyond	3.905	1.131–13.487	0.031*
**Residence**			
Rural	0.281	0.375–1.330	0.281
Urban (reference group)	1		
**Children**			
No children	3.789	1.789–8.028	0.001*
Have children (reference group)	1		
**Type of medical insurance**			
New rural cooperative medical insurance (reference group)	1		
Medical insurance for nonworking urban residents	2.767	1.171–6.535	0.02*
Medical insurance for working urban residents	11.62	1.528–88.389	0.018*
**Who will cover your medical expenses?**			
Own	2.568	1.511–4.365	< 0.001*
Children or spouse (reference group)	1		

*ADs* Advance directives, *OR* Odds ratio, *CI* Confidence interval. *p < 0.05

### End-of-life care

Table 5 shows the attitudes and preferences of patients for end-of-life care. A total of 79.43% of participants would like medical staff to discuss end-of-life arrangements with them. A total of 63.29% of patients were willing to receive end-of-life care, even though it would not delay death. Over one-half of patients with brain tumours wanted resuscitation, 27.21% did not, and 6.97% of the participants were unsure. As many as 45.45% of participants thought that they did not need life support in a persistent vegetative state, whereas 50.63% wanted life support. Furthermore, 7.92% of participants could not decide.

**Table 5:** Knowledge and preferences of end-of-life care of the study participants (*n* = 316)

Questions	Answers	n%
Would you like the medical staff to discuss your illness and end-of-life arrangements directly with you?	I would	251 (79.43%)
	I would not	65 (20.57%)
If you were terminally ill (a few weeks before death), would you prefer receiving appropriate palliative care that cannot delay death but gives comfort?	I would	200 (63.29%)
	I would not	116 (36.71%)
If you were terminally ill (a few weeks before death) and in a critical moment (e.g. cardiac arrest), would you prefer receiving resuscitation (cardiopulmonary resuscitation, electrical defibrillation, endotracheal intubation or tracheotomy)?	I would	208 (65.82%)
	I would not	86 (27.21%)
	Not sure	22 (6.97%)
If you were terminally ill (a few weeks before death) and in a persistent vegetative state (such as brain tumour progression), would you prefer life support (including nutritional support, such as tube feeding or percutaneous endoscopic gastrojejunostomy, broad-spectrum antibiotics, blood transfusions or ventilator-assisted ventilation?)	I would	131 (41.45%)
	I would not	160 (50.63%)
	Not sure	25 (7.92%)

### Predictors of preferring appropriate palliative care

Bivariate analyses identified brain primary tumours, age less than 70 years, male sex, education of junior middle school or below, having children, new rural cooperative medical insurance or medical expenses paid by their children or spouse as significant predictors of preferring appropriate palliative care. Table 6 shows the results of the binary logistic regression model.

**Table 6:** Binary logistic regression model predicting preference for receiving appropriate palliative care (*n* = 316)

Independent Predictors	OR	95%CI	*p* Value
**Type of brain tumour**			
Brain primary tumour	4.242	2.220–8.108	0.000*
Brain metastases (reference group)	1		
**Age in years**			
30–49	0.119	0.051–0.277	0.000*
50–69	0.412	0.230–0.740	0.003*
70–89 (reference group)	1		
**Sex**			
Male	1.911	1.156–3.160	0.012*
Female (reference group)	1		
**Educational qualifications**			
Junior middle school or less (reference group)	1		
Senior high school	0.376	0.213–0.665	0.001*
College or more	0.207	0.084–0.506	0.001*
**Residence**			
Rural	1.054	0.577–1.923	0.865
vUrban (reference group)	1		
**Children**			
None (reference group)	1		
Have	1.955	1.056-–3.611	0.033*
**Type of medical insurance**			
New rural cooperative medical insurance	6.435	2.065–20.050	0.001*
Medical insurance for nonworking urban residents	3.187	0.888–11.447	0.076
Medical insurance for working urban residents (reference group)	1		
**Who will cover your medical expenses?**			
Self (reference group)	1		
Children or spouse	4.131	2.531–6.744	0.000*

*OR* Odds ratio, *CI* Confidence interval. *p < 0.05

## Discussion

### Knowledge and preference of advance directives and end-of-life care

Our study found that knowledge of ADs in brain tumour patients was lower compared to a German study, considering that Germany is a developed country wherein ADs have been legal for a few years [5, 6]. In contrast, our patients with brain tumours originated from Anhui Province, China, a region with an underdeveloped economy, and ADs are far from common in China. Thus, the knowledge of ADs is rather low. However, we found that after introducing the concept of ADs to the patients, more than half of patients with brain tumours preferred to make ADs; this result is inconsistent with a previous study showing that 22.4% of patients with cancer approved of ADs [7]. This discrepancy may be attributed to the fact that in the previous study, the participants originated from different regions of China with different cultures and had different types of cancer. Considering that brain tumour patients experience compromised medical decision-making capacity due to cognitive impairment, behavioural changes, and poor communication, knowledge of ADs is important in patients with brain tumours. Therefore, we should promote the acceptance of ADs among patients with brain tumours and encourage subsequent formation of related policies and regulations related to ADs in mainland China.

The preference for end-of-life care was roughly similar to the results of a survey of 1067 adults in Hong Kong regarding their preferences for end-of-life care; the largest number of participants thought that medical staff should speak to patients directly about their situation and end-of-life arrangements as good practice, and most of the participants would prefer to receive appropriate palliative care that may not prolong their life [8]. Conversations about end-of-life arrangements with brain tumour patients are important, and effective conversation helps ensure that patients have accurate opinions about end-of-life arrangements, such as treatment plans or prognoses [9]. Medical staff should intensify propagandizing end-of-life care to allow patients with brain tumours to make appropriate decisions.

### Predictors of preferring to make advance directives

Inconsistent with a previous study indicating that knowledge of ADs is the primary predictor, in this study, the strongest predictors of ADs were receiving surgery and radiotherapy [10]. Patients with brain tumours who were receiving surgery or radiotherapy in our study would like to make ADs likely because surgical treatment or radiotherapy treatment is usually performed during the early stages of the disease, during which the patients are in a good condition, have a positive perspective of their disease and cannot fully understand their own illness. Thus, making ADs may reflect their expectations of their illnesses. A previous study suggested that ADs made by patients with tumours in the early stages cannot consistently predict what patients want at the end of their life because they may doubt the reality of their tumour diagnosis [11]. Another study suggested that patients who completely understand the role of radiotherapy and medicine are highly likely to utilize unaggressive treatments [12]. A study on glioblastoma found that the optimal time to initiate ADs is not clear, as ADs could be introduced at diagnosis, after initial surgery, during or after radiation or adjuvant chemotherapy, and/or at the time of recurrence [13]. However, another study found that the optimal timing of introducing ADs would be after chemoradiation; they thought patients were still competent early in the disease trajectory and therefore generally able to communicate their wishes [14]. Most patients have problems understanding treatment situations, choices, and risks or benefits soon after diagnosis, and approximately half are unable to participate in decisions about care in the last weeks of life [15, 16]. Continuous re-evaluation of the patient’s support needs and need for information is required because the patient’s needs change over time with disease progression [17]. Thus, identifying an optimal time for patients with brain tumours to make ADs is a challenge, and AD interviews cannot be performed at a single time point. Instead, in the future, a longitudinal study should be encouraged to ensure the authenticity and consistency of ADs.

Next, we found that educational qualifications of college or more, having medical insurance for nonworking or working urban residents and self-payment of medical expenses are predictors of AD. These independent predictors were related to education and costs. Similarly, another study found that the rate at which Chinese patients with cancer will choose artificial ventilation will increase by almost 20% if the treatment is free [18]. Another study also reported that a high proportion of Chinese patients with high educational levels had heard of ADs and wanted to make ADs [19]. In our study, patients with brain tumours who had medical insurance for nonworking or working urban residents usually lived in modern cities and had jobs. These characteristics indicated that these patients had their own income and medical insurance that could cover 95% of the medical costs of treatment. Thus, these participants preferred to make ADs because their high educational level and economic independence provided them with their own decision-making power. The advancement of ADs among patients with brain tumours in poor economic groups and groups with low educational levels should be strengthened to ensure the popularity of ADs across economic groups. We also confirmed that with the economic development of Anhui, the overall economic situation of the patient groups will improve. Thus, the application of advanced development is guaranteed.

We also found that participants who were younger, male and without children were willing to make ADs. We found that older participants were unlikely to make ADs. This result is consistent with the result of a study in Switzerland that identified the preferences and values of 50 elderly patients with cancer towards ADs; this study found that only a minority of elderly patients with cancer were prepared to put their personal wishes into writing [20]. This discrepancy might be attributed to the following: elderly participants are highly sensitive to the Confucian values of traditional Chinese culture. However, compared to elderly patients, young patients are less influenced by Confucian values and more likely to express their individual value. In Chinese culture, women are likely to defer to their husbands. Thus, being male was one predictor of making ADs in this study. Participants without children wanted to make ADs because they did not have immediate relatives to discuss their disease condition. Given that women and the elderly are reluctant to provide ADs because of Chinese Confucian culture, ADs must be popularized amongst women and the elderly to maintain patient dignity.

### Predictors of preferring receiving appropriate palliative care

Our logistic model showed that compared to participants with brain metastases, participants with primary brain tumours were more likely to prefer appropriate palliative care. This finding is one of the most important results of this study. Appropriate palliative care does not primarily aim to prolong life or cure disease but to relieve patient symptoms and to sustain or improve functioning and quality of life, and early and consistent palliative care interventions are important for ensuring quality of life [21–23]. Our patients with brain primary tumours primarily had gliomas (90.69%); the main postsurgical treatments recommended for gliomas are surgery and radiotherapy [24, 25]. Patients with primary brain tumours usually exhibit rapid progression and poor prognosis. Importantly, primary brain tumours are severe upon diagnosis, and thus patients lack a remission period wherein they can stabilize their emotions. In contrast, participants with brain metastases from lung cancer (44.34%) and breast cancer (49.13%) might have already received surgery, radiotherapy, chemotherapy, targeted therapy or immunotherapy before the emergence of brain metastases. Therefore, patients with brain metastases already understand these treatment goals and potential outcomes and are unlikely to prefer appropriate palliative care that could not prolong their life. A retrospective study indicated that there remains a wide degree of variation in the timing of end-of-life discussions, and a standard approach to end-of-life care has not yet been established [26]. There is a need to explore the preference of end-of-life care in every stage of disease progression, which may help patients with brain tumours establish a standard approach.

We found that patients with an education of junior middle school or below, new rural cooperative medical insurance (which can cover 50% of total medical expenses) and medical expenses paid by their children or spouse preferred receiving appropriate palliative care, in contrast to results of a study in Taiwan. A previous study found that patients with low socioeconomic status, metastatic malignant disease, residing in urban areas or in hospitals with more abundant health care resources were likely to receive aggressive end-of-life care to delay death [27]. However, one American study reported that patients living in low-income zip codes were less likely to receive aggressive end-of-life cancer treatment than patients living in other zip codes [28]. Aggressive end-of-life care may mean that the patient will spend high amounts of money on aggressive treatments. Patients with brain tumours with financial hardship and low educational qualifications usually cannot afford such treatments by themselves. In China, expenditure for the diagnosis and treatment of patients with cancer seems catastrophic, and cancer-related financial problems create barriers to seeking health care among survivors, so identifying cancer survivors who are more likely to experience financial hardship is important [29, 30].

In our study, we found that participants who were older, male and had children were more likely to receive appropriate palliative care; this result might imply that Confucian values still play an important role in our traditional culture [31]. In traditional Chinese culture, having the young die before the elderly is unacceptable, and many young patients with brain tumours and their family members insist on receiving aggressive treatments until the last moment of life.

Promoting the model and content of end-of-life care among patients with brain tumours and familiarising them with the results that end-of-life care could bring to them are crucial. These approaches could help patients with brain tumours further understand end-of-life care and make reasonable choices. Only in this way could patients with brain tumours avoid the side effects of excessive medical treatment and prevent family members from making the wrong decisions for patients with brain tumours after they lose the ability to speak for themselves.

### Limitations

The findings of our study should be considered in terms of several limitations. First, this was a cross-sectional study. Patients with brain tumours only received face-to-face interviews, our patients were in different stages of the disease trajectory, and the cognitive competence of their own illness condition varied. This may have influenced our findings related to the preference of preferring to make ADs and preferring to receive appropriate palliative care. These preferences may change as patients with brain tumours enter next stage of disease progression. To ensure that the results reflect what patients with brain tumours truly need, a longitudinal study should be performed to identify the optimal time for patients to state their true preferences. Next, this was a disease-specific study. We also did not ask patients with brain tumours about clinical symptoms and signs, and these patients often suffer from pain or headache, epilepsy, venous thromboembolism, fatigue, mood and behavioural disorders. Were there some connections between symptom management and the willingness to choose appropriate palliative care? Our questionnaire aimed at patients with brain tumours is not detailed or comprehensive enough to answer this question, which is a limitation of the study. In the future, a comprehensive and disease-specific questionnaire should be designed. Additionally, our study sample was limited, and our study was performed in only two hospitals and thus may generate sample bias. To reflect the knowledge and preferences of more patients with brain tumours and make our findings more convincing, an increasing number of patients with brain tumours in different hospitals should be incorporated into the study.

## Conclusions

This study illustrated the attitudes and preferences of patients with brain tumours regarding ADs and end-of-life care. Although ADs and end-of-life care have not been systematically applied in Chinese patients with brain cancer and knowledge of ADs is limited, most participants still prefer to demonstrate their individual values. Additional efforts should be made for patients with primary brain tumours undergoing surgery and radiotherapy, as well as those low socioeconomic status, and a longitudinal and comprehensive study is encouraged to promote disease-specific ADs among Chinese patients with brain tumours.

## Supplementary Information

**Additional file 1.** Details of questionnaire.

## Data Availability

The datasets used and/or analysed during the current study are available from the corresponding author on reasonable request.

## Abbreviations

ADs: Advance directives
KPS: Scores: Karnofsky scores
OR: Odds ratio
CI: Confidence interval
