# Supplementary material for: Advance directives and end-of-life care: knowledge and preferences of patients with brain Tumours from Anhui, China
Source: BMC Cancer. 2021 Jan 5;21:25. doi: 10.1186/s12885-020-07775-4 (PMC7786498; doi:10.1186/s12885-020-07775-4)
Supplement: Supplementary file 1 — Additional file 1. Details of questionnaire. [file 12885_2020_7775_MOESM1_ESM.docx]

**ADDITIONAL FILE1**

**Details of questionnaire**

Questions about ADs

1. Do you heard of advance directives (a written statement of a person's wishes regarding medical treatment, often including a living will, made to ensure those wishes are carried out and let the person be unable to communicate them to a doctor)?

Yes/No

(2) In the future, if advance directives are legal in China and you were in a condition which you cannot make your own medical decisions (e.g. coma or cannot speak), would you like to make an advance directive?

I would/I wound not

(3) If you preferred to make an advance directive, the reasons were:

1. Make sure the end of life is comfortable and avoid painful feeling;
2. Avoid burdening family economic condition;
3. Hope own wishes could be respected;
4. Avoid imposing burdens on society;
5. Consider the quality of life is more important than the length of life;
6. Religious beliefs;

g) Witness the rescue of other people.

(4) If you didn’t prefer to make an advance directive,the reasons were:

a) Family members will decide for me;

b) Let it be;

c) No need to think about it now and doctor will decide for me;

d) My decision may change;

e) Not familiar with the concept of advance directives;

f) Religious beliefs.”

Questions about End-of-Life care

Firstly, patients were introduced the concept of End-of-Life care(End-of-Life care cannot cure your illness, it is a care given to people who are near the end of life and have stopped treatment to cure or control their disease. End-of-life care includes physical, emotional, social, and spiritual support for patients and their families. The goal of end-of-life care is to control pain and other symptoms so the patient can be as comfortable as possible. End-of-life care may include palliative care, supportive care, and hospice care.

1. Would you like the medical staff to discuss your illness and end-of-life arrangements directly with you?

I would/I wound not

1. If you were terminally ill (a few weeks before death), would you prefer receiving approriate palliative care which cannot delay death but gives comfort?

I would/I wound not

1. If you were terminally ill (a few weeks before death) and in a critical moment (eg, cardiac arrest), would you prefer receiving resuscitation (cardiopulmonary resuscitation, electrical defibrillation, endotracheal intubation, tracheotomy)?

I would/I wound not/Not sure

(4) If you were terminally ill (a few weeks before death) and in a persistent vegetative state (such as brain tumor progression),would you prefer life support (including nutritional support such as tube feeding or percutaneous endoscopic gastrojejunostomy, broad-spectrum antibiotics, blood transfusions, ventilator assisted ventilation) ?

I would/I wound not/Not sure
